# Annotations

**Published:** 1894-02-03

**Authors:** 


### Annotations.

### The "Preventive" Institute: Another Complexion.

Sir Joseph Fayrer and Professor Victor Horsley tell in last week's *Nature* what they consider to be the true story of the appointment of Dr. Armand Ruffer to the directorship of the British Institute of Preventive Medicine. That story differs *toto cœlo* from the narrative put forward by Professor Roy in the previous week's issue. In one point only is Professor Roy's position untraversed. He stated that there had been no public advertisement of the new post, and therefore no attempt at selection from competing candidates. This statement is not denied. We cannot but feel that a point is scored here by the Cambridge Professor. Whatever may be the actual inwardness of the affair, it certainly would have looked better, and would have been more satisfactory to the greater world of science, if every experimental physiologist in the empire had been allowed, at least a longing glance at this new and chief "plum" of his profession. We do not suppose that any other appointment would have been made, however many candidates might have presented themselves. The really suitable candidates might have been counted on the fingers of one hand, and probably Dr. Ruffer was *facile princeps* among them. Nevertheless, we insist that it is always best in these cases to do what satisfies "form" as well as what is consonant with reason. For the rest, Professor Roy seems to have failed in frankness in his appeal to the scientific world; and Sir Joseph Fayrer and Mr. Horsley, by a plain recital of facts, have practically put him out of court.

### The Microbe or the Missionary?

In the conflict between the new ideas and ways of the West and the older ideas and ways of the East, it is most interesting to note what unexpected objects and circumstances sometimes prove to be extremely important factors. The microbe, for example, may ultimately be found more effectual as a converting agent than either the missionary or the machine gun. Mr. E. H. Hankin, of her Majesty's Indian Service, is bacteriologist to the North-West Provinces and Oude. He has just published in a contemporary a brief report on a regimental dairy in India. This is manifestly a subject of great importance, not only to our soldiers on the spot, who suffer from more or less continuous typhoid, and sometimes from alarming exacerbations of that dangerous disease, but also to their relatives at home, and to the country generally. The microbe in drinking water is a kind of ready-made measure of the possibilities of typhoid fever which may lurk in the water. Koch gives 100 microbes per cubic centimetre as the limit of safety for drinking water. Bearing in mind this figure 100, let us see what number of microbes per cubic centimetre Mr. Hankin has actually found in some of the water used for drinking and dairy purposes in connection with the East Surrey Regiment, now stationed at Agra. Certain water stated to have been drawn from a "filter" well contained, not 100, but 1,000 microbes per cubic centimetre. But this, compared with certain other water also in use, was a perfect model of purity. Some boiled water, used for scalding, that is purifying, the milk cans, showed 88,480 bacteria per cubic centimetre. The water in which "to-day's" fresh butter was kept had 215,680 bacteria per cubic centimetre; whilst the water containing "yesterday's" fresh butter showed no fewer than 435,200 microbes per cubic centimetre, all alive and eager for business. Who can wonder, after this, that our officers and troops have much more to fear from typhoid fever than from enemies of a human kind? A single fact like this proves to demonstration that the "hoary East," notwithstanding its subtlety of intellect and the "venerableness" of its civilisation, is bound either to "go down" by the sheer weight of its own invincible ignorance of science, or to "go up" by the acceptance of the new, vital, and progressive ideas of the West? Let us send Hankins by the dozen out to India, and let them all show to the natives as well as to their rulers these "object lessons" in bacteriology and typhoid. We shall then very soon find the Orient ready to consider our Western ideas more candidly.

### A Life Assurance Medical Officers' Association.

A Life Assurance Medical Officers' Association is in course of formation, and the first general meeting of its promoters was held at 20, Hanover Square on Wednesday last. There is much need for such an association, and it is to be hoped that in time order will be evolved out of the present chaos of life assurance medicine. A standard of positive and relative life value of persons offering themselves for assurance seems to be like the snakes of Iceland—it does not exist. A "life" may be "declined" at one office to-day, "accepted at an extra" at another office to-morrow, and passed as a "first class" at a third office the day after. Such a case happened within the writer's knowledge about a month ago. The proposal was for £5,000. When the proposer was first examined the medical report stated that he had heart disease, stomach disease, albuminuria, and enlarged liver. He was, of course, promptly declined. A week or two later the same proposer, who was a most sober person, was examined by an experienced assurance specialist and recommended for acceptance at a small extra: two weeks afterwards he was examined by a still more experienced assurance specialist, and passed at ordinary rates. What that proposer could have thought of "modern medical science" who shall say? One at least of these three examiners was ashamed of his profession. There are a good many things which demand the attention of the new association as well as the establishment of a workable standard of life value. The question of medical fees cannot be considered to be fixed on either a satisfactory or a logical basis. Persons assuring for over £250 are generally paid a guinea for; under this sum only half a guinea is paid for examination by many offices. But if all examined cases of less than £250 are only worth a half guinea fee, surely there should be a limit somewhere—say £5,000—beyond which cases should be worth more than a whole guinea. If it is right to contract the doctor's fee downwards, it must surely also be logical to expand it upwards. This is a part of medical polity which demands the consideration of the new association. We do not speak from the mere money-making standpoint, but from the standpoint of the status and dignity of the whole profession of medicine. We wish the new association a happy inception and a prosperous career.

